# Preventing Chronic Disease in Women of Reproductive Age: Opportunities for Health Promotion and Preventive Services

**DOI:** 10.5888/pcd9.110281

**Published:** 2012-01-12

**Authors:** Wanda D. Barfield, Lee Warner

**Affiliations:** National Center for Chronic Disease Prevention and Health Promotion, Centers for Disease Control and Prevention, Atlanta Georgia; Centers for Disease Control and Prevention, National Center for Chronic Disease Prevention and Health Promotion

Improving the health of women of reproductive age extends beyond focusing on pregnancy and birth outcomes. Approaching women's health from a life course perspective offers an opportunity to reduce overall and pregnancy-related illnesses and deaths and to eliminate disparities through enhanced health promotion and disease prevention (1).

Recent evaluations suggest that pregnancy-related illnesses and deaths resulting from chronic disease may be increasing. In 2009, the average age for mothers at first birth was 28 years, compared with 21.4 years in 1970 (2). Older women have an increased prevalence of chronic medical conditions, leading to higher risk of adverse pregnancy outcomes. According to a nationally representative study examining trends in US hospitalizations from 1995-2006, the severity of chronic heart disease among women hospitalized during pregnancy may have increased (3). Additionally, data from the Pregnancy Mortality Surveillance System (PMSS) of the Centers for Disease Control and Prevention (CDC) (www.cdc.gov/reproductivehealth) indicate shifts in the proportion of maternal deaths from traditional direct causes of maternal deaths (eg, caused by hemorrhage or infection) toward more chronic conditions, particularly cardiovascular diseases (4). Women of reproductive age also are experiencing increases in the prevalence of chronic disease–related risk factors such as obesity, diabetes, high cholesterol, and asthma (5). Despite these increases, prevention opportunities exist to improve women's health during their reproductive years and beyond and to improve the health of future generations.

In the November 2011 issue of *Preventing Chronic Disease* (*PCD*), articles by Farr et al (6), Tong et al (7), Hayes et al (8), and Amparo et al (9) highlighted the prevalence of chronic disease conditions and risk factors that affect the health of women of reproductive age, including behavioral and environmental factors such as smoking, alcohol use, depression, physical inactivity, and lack of access to health services. Additionally, Farr et al (10), Tovar et al (11), Robbins et al (12), and Zera et al (13) summarized various chronic disease screening recommendations and interventions targeted to reach women of reproductive age and improve their health throughout life.

Health promotion for women of reproductive age includes increasing epidemiology capacity at the state, local, territorial, and tribal level to effectively use data related to chronic disease prevalence and risk. As an example, as part of the CDC Maternal and Child Health Epidemiology Program, researchers assigned to state health departments collaborate in maternal and child health and chronic disease programs in a partnership with states and the Maternal and Child Health Bureau in the Health Resource Services Administration. In the November 2011 issue of *PCD*, Hayes et al (8) and Cheng and Patel (14) illustrated well the importance of building capacity at county and state health department and even clinic levels to address chronic disease prevention in women of reproductive age.

The US Department of Health and Human Services adoption of the Institute of Medicine's recommendations of preventive services for women (www.iom.edu/reports/2011/clinical-preventive-services-for-women-closing-the-gaps.aspx) provides an opportunity to further extend clinical guidance for women's reproductive and wellness health screening through the Affordable Care Act. These recommendations provide no-cost coverage for 1) an annual well-woman preventive health visit, including preconception care, and additional visits depending on a woman's health status, needs, and other risk factors; 2) an array of contraceptives approved by the Food and Drug Administration; 3) human papillomavirus testing as part of cervical cancer screening; 4) annual counseling for sexually transmitted infections and screening for human immunodeficiency virus in sexually active women; 5) screening for gestational diabetes in pregnant women; and 6) comprehensive support and counseling for breastfeeding.

Adapted from earlier released guidelines of the World Health Organization (15), the *U.S. Medical Eligibility Criteria for Contraceptive Use* (16) has been adopted by public health and clinical organizations such as the American College of Obstetricians and Gynecologists (17). In 2010, CDC developed national guidelines that provide evidence-based information on safe and effective options for contraception for various medical conditions affecting US reproductive-aged women, including teenagers. Safe and effective use of and ready access to contraception may optimize a woman's health by providing additional time to address a preexisting health condition or risk factor (eg, diabetes, hypertension, smoking) before pregnancy and by preventing unintended pregnancy and adverse pregnancy outcomes in women with poor health.

CDC's Pregnancy Risk Assessment Monitoring System (PRAMS) (www.cdc.gov/prams), an annual survey of women with a recent live birth, provides coverage of nearly 80% of births nationwide and monitors not only trends in certain chronic diseases (eg, diabetes, hypertension, postpartum depressive symptoms) but also trends in key risk behaviors underlying many chronic diseases (eg, obesity, tobacco use, physical inactivity). PRAMS data on preconception and interconception care indicators can help programs anticipate prevention needs (18). For example, findings from Louisiana PRAMS on the prevalence of chronic disease risk factors among women of reproductive age influenced the development and implementation of a preconception health awareness project, The Stork Reality. This project targeted women who do not have regular medical homes to provide preconception and interconception health services (www.storkreality.com). Surveillance of state health policies (eg, state tobacco control policies, smoke-free air laws, spending, taxes on cigarettes) can also be evaluated with PRAMS data to examine their effect on health outcomes before, during, and after pregnancy.

A key strategy to improve the health of reproductive-aged women is to improve their continuity of care beyond pregnancy. Reproductive and postpartum health visits offer opportunities for providers to promote preventive care, screen for chronic diseases, and provide referrals to appropriate interventions. Further research is needed to evaluate the feasibility and effectiveness of interventions initiated during reproductive and postpartum health care visits, such as those that aim to reduce smoking, obesity and overweight, hypertension, high cholesterol, and diabetes (eg, screening for chronic diseases and associated risk factors during family planning or sexually transmitted disease clinic visits).

Through existing funding initiatives and new funding opportunities from the Affordable Care Act, approaches for broad public health interventions can improve the health of women of reproductive age by supporting activities that address social policies, systems, and practices that improve population health. A public health approach to chronic disease prevention through community-based prevention efforts can help address health promotion among women of reproductive age.
